# Urinary estrogen metabolites and prostate cancer: a case-control study and meta-analysis

**DOI:** 10.1186/1756-9966-28-135

**Published:** 2009-10-08

**Authors:** Maddalena Barba, Li Yang, Holger J Schünemann, Francesca Sperati, Sara Grioni, Saverio Stranges, Kim C Westerlind, Giovanni Blandino, Michele Gallucci, Rossella Lauria, Luca Malorni, Paola Muti

**Affiliations:** 1 Department of Epidemiology, National Cancer Institute Regina Elena, Rome, Italy; 2 Department of Environmental, Agricultural and Occupational Health, College of Public Health, University of Nebraska Medical Center, Omaha, Nebraska, USA; 3 Department of Medicine, State University of New York at Buffalo, Buffalo, New York, USA; 4 Department of Clinical Epidemiology and Biostatistics, McMaster University, Hamilton, Ontario, Canada; 5 Nutritional Epidemiology Unit, National Cancer Institute, Milan, Italy; 6 Health Sciences Research Institute, Warwick Medical School, Coventry, UK; 7 Division of Endocrinology, Metabolism & Diabetes, University of Colorado at Denver and Health Sciences Center, Denver, Colorado, USA; 8 Translation Oncogenomics Unit/Lab B, National Cancer Institute Regina Elena, Rome, Italy; 9 Department of Urology, National Cancer Institute Regina Elena, Rome, Italy; 10 Division of Medical Oncology, Department of Molecular and Clinical Endocrinology and Oncology, University of Naples Federico II, Naples, Italy; 11 Lester and Sue Smith Breast Center, Baylor College of Medicine, Houston, Texas, USA; 12 Scientific Direction, National Cancer Institute Regina Elena, Rome, Italy

## Abstract

**Objective:**

To investigate prostate cancer (Pca) risk in relation to estrogen metabolism, expressed as urinary 2-hydroxyestrone (2-OHE1), 16α-hydroxyestrone (16α-OHE1) and 2-OHE1 to 16α-OHE1 ratio.

**Methods:**

We conducted a case-control study within the Western New York Health Cohort Study (WNYHCS) from 1996 to 2001. From January 2003 through September 2004, we completed the re-call and follow-up of 1092 cohort participants. Cases (n = 26) and controls (n = 110) were matched on age, race and recruitment period according to a 1:4 ratio. We used the unconditional logistic regression to compute crude and adjusted odds ratios (OR) and 95% confident interval (CI) of Pca in relation to 2-OHE1, 16αOHE1 and 2-OHE1 to 16α-OHE1 by tertiles of urine concentrations (stored in a biorepository for an average of 4 years). We identified age, race, education and body mass index as covariates. We also conducted a systematic review of the literature which revealed no additional studies, but we pooled the results from this study with those from a previously conducted case-control study using the DerSimonian-Laird random effects method.

**Results:**

We observed a non-significant risk reduction in the highest tertile of 2-OHE1 (OR 0.72, 95% CI 0.25-2.10). Conversely, the odds in the highest tertile of 16α-OHE1 showed a non-significant risk increase (OR 1.76 95% CI 0.62-4.98). There was a suggestion of reduced Pca risk for men in the highest tertile of 2-OHE1 to 16α-OHE1 ratio (OR 0.56, 95% CI 0.19-1.68). The pooled estimates confirmed the association between an increased Pca risk and higher urinary levels of 16α-OHE1 (third vs. first tertile: OR 1.82, 95% CI 1.09-3.05) and the protective effect of a higher 2-OHE 1 to 16α-OHE1 ratio (third vs. first tertile: OR 0.53, 95% CI 0.31-0.90).

**Conclusion:**

Our study and the pooled results provide evidence for a differential role of the estrogen hydroxylation pathway in Pca development and encourage further study.

## Introduction

Prostate cancer (Pca) is the most frequently diagnosed malignancy and the second leading cause of cancer death among men in Western countries [1]. Notwithstanding the importance of this tumor, its causes remain largely unknown. Age, family history, race and country of residence are the only established risk factors, but they explain only a small proportion of Pca incidence [2].

A considerable number of studies have addressed prostate sensitivity to androgens in relation to outcomes varying from normal prostate growth to benign and malignant diseases [3-5]. However, the role played by estrogens in the pathogenesis of a wide spectrum of prostate physiologic and pathologic conditions is drawing increasing attention [6]. In regards to Pca, experimental data from studies conducted in Noble (NBL) rats strongly suggest a critical role for estrogens in prostate carcinogenesis. Indeed, in NBL rats chronically treated with testosterone, the addition of estrogens is associated with a 100% incidence of prostate adenocarcinomas, whereas the administration of testosterone as a single agent produces Pca in approximately 30 to 40% of treated animals [7,8]. The estradiol plus testosterone treatment also induces acinar lesions that are similar to human prostatic intraepithelial neoplasia, a well recognized pre-invasive stage of adenocarcinoma [9].

Evidence is also mounting regarding the contribution of hydroxylated metabolites of estrone (E1) and estradiol (E2) to the overall estrogenic activity. The mutually exclusive hydroxylation of E1 and E2 at positions C-16α or C-2 leads to the production of either biologically active estrogens (16α-hydroxyestrone/estradiol) or derivatives with virtually no estrogenic activity (2-hydroxyestrone/estradiol), respectively [10-12]. The different profiles in terms of biological activity and genotoxic properties might have consequences in terms of Pca risk.

However, the overall body of evidence remains particularly limited when considering estrogen metabolites in relation to Pca risk. Our prior case-control study, conducted in Buffalo, NY, suggested an increased risk of clinically evident Pca in men with a lower 2-OHE1/16α-OHE1 ratio [13]. Similar results from studies evaluating breast cancer, as another hormone-dependent tumor, support this observation [14-18].

In the current case-control study, we have further tested the hypothesis that the pathway favoring 2-hydroxylation over 16α-hydroxylation is associated with a reduction in Pca risk. We also conducted a systematic review of the literature to evaluate the totality of the evidence of this research question.

## Material and methods

From 1996 to 2001, 1961 men were enrolled in the Western New York Health Cohort Study (WNYHCS). A detailed description of the WNYHCS study design, methods and participants' characteristics is available elsewhere [14].

In brief, all participants provided informed consent; the Human Subjects Review Board of the University at Buffalo, School of Medicine and Biomedical Science approved procedures for protection of human subjects in the study. At the time of recruitment, trained interviewers collected extensive data on demographics and life style during in-person interviews. The use of a standardized protocol allowed for the collection of anthropometric data. The study participants donated morning spot urine which was kept at -80°C until biochemical determinations.

From January 2003 through September 2004, we completed the Western New York Health Cohort (WNYHC) re-call and follow-up. For the purposes of the present case-control study (PROMEN II study), the re-call process included male participants who met the following inclusion criteria: age at recruitment between 50 and 85, baseline history negative for malignancies, cardiovascular diseases and clinically defined type-2 diabetes. On this basis, the re-call and follow-up process involved 1092 cohort participants. Among them, 52 were not eligible for medical reasons other than Pca, 46 had died from causes other than Pca, 22 had moved out of Erie and Niagara Counties, and 117 were not able to be contacted by mail or phone. Among the remaining 855 study participants, 232 refused to join the study, 40 were scheduled but cancelled the appointment and 8 were still in-course of assessment at the end of the follow-up period. In this group of non-participating subjects, all of the cohort members referred to being free from Pca in their telephone interviews. Thus, 575 participants joined the study, accounting for an overall participation rate of 67% (575/855).

Pca cases were men who had been diagnosed with incident, histologically confirmed Pca within the time-frame between their recruitment in the WNYHC and the end of the follow-up period. Identifying Pca cases was based on the participants' reports at the re-call, which was subsequently validated by clinical records provided by their urologists. We identified and validated a total number of 41 incident prostate cancer cases. The 534 control subjects were male members of the WNYHC who, based on their report, were free from clinically evident Pca at the time of diagnosis of the related case. The control status was validated with a serum PSA assessment on a blood sample donated at the time of recall. We used a PSA cut-off value of 4 ng/ml [15]. Among the study participants whose PSA level was higher than 4 ng/ml, we ultimately included in the control group only those who tested negative at the prostate biopsy. We requested and obtained the pertinent medical records from the urologists.

For each case, four control subjects were randomly chosen after matching for age (within a 3-year-range), race and date of recruitment. The independent variables of interest, namely 2-OHE1, 16α-OHE1 and the 2-OHE1 to16α-OHE1 ratio, were available for 110 controls and 26 cases, thus we conducted the present analysis on 136 subjects.

### Hormonal Determinations

For standardization purposes, we collected morning spot urine between 7:00 a.m. and 9:00 a.m. from all participants. We then transferred the aliquoted urine samples to the Eppley Institute, University of Nebraska Medical Center (UNMC), and stored them at -80°C until analysis. Each sample was thawed only once prior to analysis. We handled urine samples identically and located them in the laboratory runs randomly. All laboratory personnel were blinded in regards to case-control status. All of the study samples were analyzed in duplicate. Two-milliliter aliquots of urine were partially purified throughout solid phase extraction (SPE) with a phenyl cartridge (Varian, Palo, Alto, CA) and ultra-performance liquid chromatography/tandem mass spectrometry (LC/MS-MS). Analytes were identified based on their retention time and tandem mass spectrometry. Standards of the catechol estrogens 2-OHE_1_(E_2_) and 16α-OHE_1_(E_2_) were purchased from Steraloids Inc. (Newport, RI).

To avoid the artifacts and errors introduced by maintaining the urine samples at 37°C for 8 hours, we carried out all the analyses without glucuronidase/sulfatase treatment. We adjusted urine samples to pH 7 with 1 M NaOH or 1 M HCl.

We performed the LC/MS analyses through a Waters Acquity ultra-performance liquid chromatography (UPLC) system connected with a high performance Quattro Micro triple quadruple mass spectrometer designed for LC/MS-MS operation. We performed the analytical separations on the UPLC system using an Acquity UPLC BEH C18 1.7 μm column (1 × 100 mm) at a flow rate of 0.15 ml/min. We then moved the elutions from the UPLC column to the Quattro Micro mass spectrometer.

The ionization method used for MS analysis was Electrospray ionization (ESI) in both the positive ion (PI) and negative ion (NI) mode with an ESI-MS capillary voltage of 3.0 kV, an extractor cone voltage of 3 V, and a detector voltage of 650 V. We performed the MS-MS in the multiple reaction monitoring (MRM) mode to produce structural information about the analytes by fragmenting the parent ions inside the mass spectrometer and identifying the resulting daughter/fragment ions. We processed the resulting data and quantified the estrogen metabolites using the QuanLynx software (Waters).

To calculate limits of detection, we injected various concentrations of the analytes to LC/MS-MS. The detection limit was considered to be the injected amount that resulted in a peak with a height at least two or three times higher than the baseline. The detection limits of 2-OHE_1 _and 16α-OHE_1 _were 18 fmol and 349 fmol, respectively. Intra-assay coefficients of variation for 2-OHE_1 _and 16α-OHE_1 _were 3.2% and 3.0%, respectively. Inter-assay coefficients of variation were 1.9% and 3.5%, respectively.

We had previously measured the intra- and inter-individual variability for 2-OHE1, 16α-OHE1 determinations and their ratio over a one year period [13]. The intra-class correlation coefficients (ICCs) and lower limit of 95% CI (in parentheses) were 0.70 (0.46), 0.63 (0.35) and 0.78 (0.62), respectively. We had previously provided a detailed description of the procedures related to the reliability assessment [13].

### Systematic Review

We conducted a systematic search of the literature to identify additional studies published up to August 2009 which examined the association between estrogen metabolites and Pca risk using our standard methods [19-22]. We searched MEDLINE (January 1966 onwards) and EMBASE (January 1980 onwards). An expert librarian designed a search strategy combining terms for estrogens, estrogen metabolites and prostate specific antigen (PSA) with terms for Pca (available upon request). We screened titles and abstracts in duplicate using the following inclusion criteria: observational studies investigating prostate cancer risk in relation to estrogen metabolism. We included studies providing at least one measure of either urinary or circulating levels of 2-OHE1, 16α-OHE1 and the 2-OHE1 to 16α-OHE1 ratio.

### Statistical analysis

We examined distributions for all variables of interest by determining the frequencies, mean, median and measures of variance. To evaluate the statistical significance of the unadjusted associations between case/control status and participants' characteristics, we used either Fisher's exact tests or Pearson's chi-square tests for categorical variables.

The 2-OHE1 and 16-αOHE1 urinary levels were standardized by total urinary creatinine. We used unconditional logistic regression to compute crude and adjusted odds ratios (OR) and 95% confident interval (CI) of Pca in relation to 2-OHE1, 16-αOHE1 and the ratio of 2-OHE1 to 16α-OHE1 by tertiles of urine concentrations. We used the same models to test for significance in trends of association for any of the independent variables. We computed the cut-off points of the previously mentioned tertiles based on the distributions of estrogen metabolites in control subjects. We analyzed each independent variable separately. Based on the published literature, we identified age, race, education level, BMI and waist-to-hip ratio as possible covariates and tested them using regression models. Although none of them was a confounder for the investigated associations, we included age in years in further analyses based on its biological relevance in prostate carcinogenesis [2].

We verified several sources of potential bias. Because the exclusion of participants with missing data for any of the two outcome variables could have introduced a source of bias in our final sample, we examined data by subsets. Each of the two datasets included men with no missing data for either urinary levels of 2-OHE1 or 16-αOHE1. We then examined by case-case and control-control comparing the characteristics of the 136 subjects (110 controls and 26 cases) with no data missing for any of the considered variables and those of the subjects (534 controls and 41 cases) who fulfilled our study eligibility criteria. Finally, we compared the subjects in the latter category [575] to the 517 original cohort members who did not join the study either because they did not fulfil the inclusion criteria, were lost to follow-up or were not willing to participate.

To date, no data exists related specifically to any of these three categories (i.e. co-morbidity data pertinent to the WNYCS). Thus, we considered these 517 male subjects as part of an overall, although heterogeneous, category. As expected, the 517 males from the original cohort who did not ultimately join our study showed statistically significant differences when compared to the 575 included study participants. We analyzed these data using SPSS version 14.0 (SPSS, Inc., Chicago, IL).

### Meta-analysis

We planned to combine the results from the current study with those identified in the systematic review using the DerSimonian-Laird random effects method expressing the pooled estimates in terms of summary OR and 95% CI. We calculated I^2 ^to assess heterogeneity across study results applying the following interpretation for I^2 ^(J Higgins, personal communication): 0-50 = low; 50-80 = moderate and worthy of investigation; 80-100 = severe and worthy of understanding; 95-100 = aggregate with major caution [23]. We used Revman 5.0 for the meta-analysis (Copenhagen: The Nordic Cochrane Centre, The Cochrane Collaboration, 2008).

## Results

Table 1 shows the descriptive characteristics of the study participants. No significant differences emerge when comparing cases and controls by age, race, education, and anthropometrics.

**Table 1:** Participants Descriptive Characteristics by Case-Control Status, PROMEN Study, 1996-2001

		**Prostate Cancer**				
		**Control**		**Case**		**two-tails**
		**n**	***%***	**n**	***%***	**p-value**
		
		110	*80.88*	26	*19.12*	
**Age**						
	***50-59***	31	*28.20*	7	*26.90*	
	***60-69***	40	*36.40*	9	*34.60*	
	***70-79***	39	*35.50*	10	*38.50*	
						0,902
					
**Race**						
	***Black***	4	*3.60*	1		
	***White***	106	*96.0*	25		
						1.000
					
**Years of Education**						
	***8-13***	66	*60.00*	16	*61.50*	
	***14-18***	44	*40.00*	10	*38.50*	
						1.00
					
**BMI**						
	***≤ 25***	25	*22.90*	6	*23.10*	
	***25-30***	55	*50.50*	11	*42.30*	
	***≥ 30***	29	*26.60*	9	*34.60*	
						0.683
					
**Waist circumference**						
	***≤ 97,50***	56	*51.40*	10	*38.50*	
	***> 97,50***	53	*48.60*	16	*61.50*	
						0.279
					
**Hip circumference**						
	***≤ 102,50***	56	*51.40*	12	*46.20*	
	***> 102,50***	53	*48.60*	14	*53.80*	
						0.668
**Waist to hip ratio**						
	***≤ 0,95***	55	*50.50*	14	*56.00*	
	***> 0,95***	54	*49.50*	11	*44.00*	
						0.662

*BMI: body mass index expressed as weight in kilograms divided by the square of height in meters (kg/m^2^)

In Table 2, we report crude and age-adjusted Pca risk estimates in relation to tertiles of urinary estrogen metabolites and their ratio. The OR in the highest compared to the lowest tertile of 2-OHE1 was 0.72 (95% CI 0.25-2.10). Conversely, the odds in the highest tertile of 16α-OHE1 was 1.76 (95% CI 0.62-4.98). Finally, the 2-OHE1 to 16α-OHE1 ratio showed a non-significant risk reduction across tertiles (OR 0.56, 95% CI 0.19-1.68, in the highest tertile). When we tested the independent variables of interest for significance in trends of associations, none of the models produced significant results.

**Table 2:** Crude and Adjusted Prostate Cancer Risk Estimates

			**Cs/Co**^a^	**Crude OR**^b^	**95% CI**^c^	**Adjusted OR**^d^	**95% CI**^c^
**2OHE1**							
	***1st tertile***	***≤ 0.21***	10/37	1	-	-	-
	***2nd tertile***	***0.21 - 2.26***	9/37	0.90	0.33 -2.47	0.90	0.32-2.46
	***3rd tertile***	***> 2.26***	7/36	0.72	0.25 -2.10	0.69	0.23-2.03
	***trend***			0.85	0.50-1.44	0.83	0.49-1.42
	***P for trend***			*0.55*		*0.50*	
			
**16OHE1**							
	***1st tertile***	***≤ 61.84***	7/37	1	-	-	-
	***2nd tertile***	***61.84 - 158.74***	7/37	1.00	0.32 - 3.13	1.00	0.32-3.13
	***3rd tertile***	***>158.74***	12/36	1.76	0.62 - 4.98	1.73	0.58-5.14
	***trend***			1.35	0.80-2.30	1.33	0.76-2.33
	***P for trend***			*0.26*		*0.31*	

**2OHE1/16OHE1**							
	***1st tertile***	***≤ 0,31***	11/37	1	-		-
	***2nd tertile***	***0.31-1.64***	9/37	0.82	0.30-2.21	0.80	0.30-2.17
	***3rd tertile***	***> 1.64***	6/36	0.56	0.19 - 1.68	0.57	0.19-1.71
	***trend***			0.75	0.44-1.29	0.76	0.44-1.30
	***P for trend***			*0.30*		*0.31*	

^a ^matched on age, race and recruitment period

^b ^odds ratio; ^c^95% confidence interval; ^d^adjusted for age

Analyzing data by subsets including only one of the two outcome variables did not affect the study results at any level. From the case-case and control-control comparison, no significant differences emerged between the participants who had been included in the present analyses and those who had been excluded because of missing data items.

### Results of the systematic review

Our search of the literature yielded a total of 289 unique citations. Based on the titles and abstracts screening of the retrieved citations, only our previously conducted case-control study [13] and the study from Yang and colleagues [24] met the eligibility criteria. Unfortunately, we could not include the latter manuscript in our meta-analysis. In the study from Yang et al the whole control group, which itself represents the vast majority of the overall sample (118/139), is part of the Western New York Health Cohort and directly stems from the recall process carried out between January 2003 and September 2004 as part of the PROMEN II study. The inclusion of this study would artificially inflate the size of our meta-analysis and potentially bias our results. Thus, only another study, namely our previously conducted case-control study, was included in our meta-analysis.

**Figure 1:**
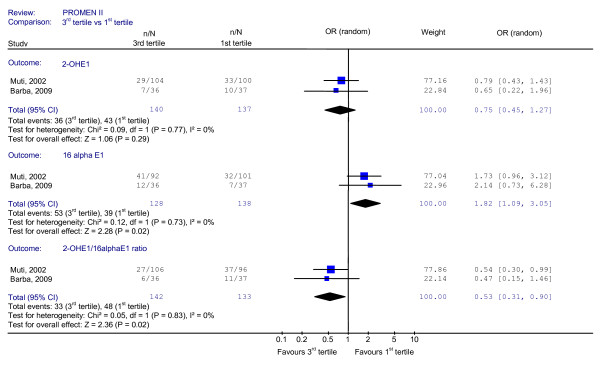
**Pooled estimates of Prostate Cancer Risk in relation to Estrogen Metabolites**.

Figure 1. shows the results of the meta-analysis results. The pooled data are based on 122 Pca patients and 414 controls. The meta-analysis suggested an association between an increased Pca risk and higher urinary levels of 16α-OHE1 (third vs. first tertile: OR 1.82, 95% CI 1.09-3.05) and the protective effect of a higher 2-OHE 1to16α-OHE1 ratio (third vs. first tertile: OR 0.53, 95% CI 0.31-0.90). We found no statistically significant results for 2-OHE1. There was no evidence of heterogeneity (I^2 ^= 0, for any of the reported estimates).

## Discussion

The results of this study and meta-analysis suggest that the metabolic pathway favoring 2-hydroxylation over 16α-hydroxylation might be associated with a reduction in Pca risk. While the findings from this case-control study are not statistically significant, they appear consistent with those from a previously conducted, larger case-control study on the protective role of hydroxylated metabolites with virtually no estrogenic activity in the development of Pca [13]. A meta-analysis of the results from these two studies, preceded by a systematic search of the literature showing no additional studies, revealed evidence in support of the study hypothesis.

Our study has several strengths. The prospective design allowed for sample collection years before Pca diagnosis. On this basis, it is plausible that the observed differences in urinary levels of estrogen metabolites by case-control status were not biased by any cancer-related hormonal activity in the diseased subjects group. In theory, the long-term effects of cryopreservation represent a potential source of variability because of the occurrence of sample degradation, but data from a previously conducted prospective study showed stability of estrogen metabolites over time [16]. However, if any effect from degradation exists, it should be similar for cases and controls because of the matching for date at recruitment. At the time of the WNYHC recall, we tested control subjects for a potential presence of latent prostate cancer by serum analysis for PSA and, for those men whose PSA value exceeded the pre-defined cut-off, by prostate biopsy. This approach increases our confidence in the case-control definition and reduces the possibility for misclassification bias. We adopted several strategies to control for potential sources of hormone variability. In conducting the WNYHC recruitment and recall, we applied inclusion criteria requiring the absence of pathologic conditions altering hormone metabolism (i.e. type 2 diabetes). We observed highly standardized conditions at sample collection, handling and assaying. All hormone determinations were performed at the end of the study, to reduce technical variability. We also evaluated the intra-individual variability of 2-OHE1, 16αOHE1 and their ratio in a previously conducted study [13]. The resulting intra-class correlation coefficients (ICC) indicated high reliability, thus reducing the chance that a measurement error might have affected the study results to a significant extent.

Our study also has several limitations. The sample size was very small, especially for cases, and none of the provided estimates reached statistical significance in the original study. The small sample size might have limited our ability to detect the investigated associations. Selection bias is another source of possible concern for several reasons. First, the participation rate was quite low (67%) and unfortunately we had limited information allowing a comparison between participating and non-participating subjects. Indeed, the lack of mortality or co-morbidity data prevented us from characterizing those members of the original cohort who were excluded because of diseases other than Pca or death. The final comparison between the 575 men who joined the study and the 517 cohort members who did not show significant differences. The exclusion of participants with missing data either for any of the outcome variables or any of the considered variables represents an additional, potential source of bias. Neither the analyses conducted by subsets including only one of the outcome variables, nor the analyses performed by case-case and control-control comparison between subject with and without missing data items showed significant results.

We conducted a systematic search of the literature and combined the available results in a meta-analysis. We found significant evidence supporting the protective role of the metabolic pathway favoring 2-hydroxylation over 16α-hydroxylation in Pca development. This increases our confidence in the single studies' results, which were consistent, and might indicate that the lack of significance was mainly due to the limited sample size of the single studies.

Despite their historical use in prostate cancer treatment, our knowledge regarding the effects of estrogens on prostate, their role in cancer development and the mechanisms mediating their action as therapeutic agents is quite limited. The published literature mainly focuses on the effects of circulating estrone and estradiol in relation to prostate cancer risk, providing inconsistent evidence [17,18,25,26]. A wide variety of methodological issues ranging from the restricted sample size to possible bias introduced by uncontrolled sources of hormonal variability might provide a partial explanation to the cited inconsistency. It is also plausible that the surmised exposures have not been captured over periods comparable by degree of prostate sensitivity to hormonal influences across the different studies. The lack of consideration for factors potentially relevant to the overall estrogenic activity, namely, hydroxylated metabolites of E1 and E2, might provide a further explanation that would integrate the aforementioned hypotheses.

The dominating hydroxylation pathway significantly affects the biological activity of estrogen metabolites. Indeed, 16α-OHE1 binds with high affinity the estrogen receptor and exerts a strong estrogenic action that leads to increased cell proliferation and DNA synthesis [27,28]. Conversely, 2-OHE1 exerts a weak agonist effect on the oestrogen receptor and shows anti-angiogenic properties [29,30].

Little epidemiologic evidence exists with regard to the hypothesis investigated in the present study. Our previous study results support the association between elevated 2-OHE1 urinary levels and a reduced Pca risk (OR 0.83 95% CI 0.43-12.44), whereas elevated16α-OHE1 urinary levels are associated with increased risk (OR 1.69 95% CI 0.93-3.06, p for linear trend 0.002) [13]. In their case-control study, Yang and colleagues found no significant difference in the median levels of 2-OHE1 and 16α-OHE between the compared groups. However, the sample size was very limited and the number of cases extremely low [24]. In their cross-sectional study, Teas et al evaluated the variability of the urinary levels of 2-OHE1 and 16αOHE1 in a sample of African-American men attending prostate cancer screening clinics and investigated any possible relation of these two metabolites with PSA. They reported an overall significant reduction in 2-OHE1 per each 1.0 ng/ml increase in PSA [31].

Further evidence of the role of sex steroid hormones in prostate cancer emerges from studies focusing on the role played by estrogen metabolites in breast carcinogenesis. Several case-control and cohort studies show that women who metabolize a larger proportion of estrogens via the 16α-hydroxy pathway may be at a significantly higher risk of breast cancer compared to women who metabolize proportionally more estrogens via the 2-hydroxy pathway [16,32-34]. We observed a 40% breast cancer risk reduction in women whose 2-hydroxyestrone (2-OHE1) to 16α-hydroxyestrone ratio was in the highest tertile of the distribution compared to those in the lowest tertile [35].

In summary, in the context of a still limited scientific evidence base, our study and meta-analysis provide data supporting a differential role of the estrogen hydroxylation pathway in prostate cancer development. The small sample size of our original study prevents us from drawing strong conclusions, but the results of our meta-analysis including the second study provide us with greater evidence in support of the investigated association and the need for further studies.

## Competing interests

The authors declare that they have no competing interests.

## Authors' contributions

MB contribution to data analysis, results interpretation, manuscript drafting, review coordination

LY laboratory assays

HJS methodological advice, critical revision of the manuscript, systematic review conception

FS and SG data analysis

SS, KW, GB, MG critical revision of the manuscript

PM case-control study conception and design, methodological advice, critical revision of the manuscript

All authors have read and approved the final version of the manuscript
